# Skeletal muscle secretome in Duchenne muscular dystrophy: a pivotal anti-inflammatory role of adiponectin

**DOI:** 10.1007/s00018-017-2465-5

**Published:** 2017-02-10

**Authors:** S. Lecompte, M. Abou-Samra, R. Boursereau, L. Noel, S. M. Brichard

**Affiliations:** Endocrinology, Diabetes and Nutrition Unit, Institute of Experimental and Clinical Research, Catholic University of Louvain, UCL/EDIN B1.55.06 – Av. Hippocrate, 55B-1200, Brussels, Belgium

**Keywords:** ApN, Inflammation, DMD, Human myotubes, Muscle secretion

## Abstract

**Background:**

Persistent inflammation exacerbates the progression of Duchenne muscular dystrophy (DMD). The hormone, adiponectin (ApN), which is decreased in the metabolic syndrome, exhibits anti-inflammatory properties on skeletal muscle and alleviates the dystrophic phenotype of mdx mice. Here, we investigate whether ApN retains its anti-inflammatory action in myotubes obtained from DMD patients. We unravel the underlying mechanisms by studying the secretome and the early events of ApN.

**Methods:**

Primary cultures of myotubes from DMD and control patients were treated or not by ApN after an inflammatory challenge. Myokines secreted in medium were identified by cytokine antibody-arrays and ELISAs. The early events of ApN signaling were assessed by abrogating selected genes.

**Results:**

ApN retained its anti-inflammatory properties in both dystrophic and control myotubes. Profiling of secretory products revealed that ApN downregulated the secretion of two pro-inflammatory factors (TNFα and IL-17A), one soluble receptor (sTNFRII), and one chemokine (CCL28) in DMD myotubes, while upregulating IL-6 that exerts some anti-inflammatory effects. These changes were explained by pretranslational mechanisms. Earlier events of the ApN cascade involved AdipoR1, the main receptor for muscle, and the AMPK-SIRT1-PGC-1α axis leading, besides alteration of the myokine profile, to the upregulation of utrophin A (a dystrophin analog).

**Conclusion:**

ApN retains its beneficial properties in dystrophic muscles by activating the AdipoR1-AMPK-SIRT1-PGC-1α pathway, thereby inducing a shift in the secretion of downstream myokines toward a less inflammatory profile while upregulating utrophin. ApN, the early events of the cascade and downstream myokines may be therapeutic targets for the management of DMD.

## Introduction

Duchenne muscular dystrophy (DMD) is an X-linked muscle disease, with a prevalence of 1 in 3500 boys worldwide. Dystrophin deficiency is the primary defect in DMD. This protein is an essential component of the sarcolemmal dystrophin-associated glycoprotein complex, which is a linkage between the muscle cell cytoskeleton and the extracellular matrix. Disturbance of this complex leads to sarcolemmal instability and increased vulnerability to mechanical stress [1]. Contraction of dystrophin-deficient myofibers produces severe damage and generates cycles of muscle fiber necrosis and regeneration [2]. These alterations lead to chronic inflammation, which is a crucial feature of the pathogenesis of this disease [3]. In this context, cytokines and chemokines play an important and diversified role in DMD-associated muscle inflammation.

Skeletal muscle has been identified as an endocrine organ that has the capacity to produce and secrete myokines. The muscle secretome consists of several hundred cytokines or peptides, which may exert local effects (via an autocrine or paracrine mode) or systemic effects (via an endocrine mode). These myokines may mediate immune and metabolic responses [4, 5]. In the past few years, some reports have analyzed muscle secretome profiling in rodent [6, 7] and human [8–11] models. Only one work has focused on the secretome profiling of dystrophic myotubes, originating from mdx mice (a model of DMD) [12]. In the present study, we focus on the myokine secretion profile of human dystrophic muscle cells.

Adiponectin (ApN) is a hormone abundantly secreted by adipocytes under normal conditions. ApN exerts pleiotropic actions promoting insulin-sensitizing, fat-burning, and anti-inflammatory properties as well as modulatory effects on oxidative stress, thereby thwarting simultaneously several facets of the metabolic syndrome [13, 14]. Adiponectin receptor 1 (AdipoR1) and 2 (AdipoR2) serve as major receptors for ApN, with AdipoR1 being mainly expressed in skeletal muscle [15].

ApN attenuates inflammatory and oxidative responses to multiple stimuli by modulating different signaling pathways in a variety of cell types [16]. The pathway including AMPK, a deacetylase, Sirtuin (SIRT) 1, and the peroxisome proliferator-activated receptor-γ coactivator-1α (PGC-1α) is required for the beneficial effects of ApN on the skeletal muscle [17, 18].

Recently, we evidenced that ApN is an extremely powerful hormone that protects mouse muscle against inflammation and oxidative stress, even when the abnormalities are extremely severe and long-lasting such as in mouse dystrophic muscle [18]. Moreover, we showed that mdx transgenic mice overexpressing ApN exhibited higher global muscular force and endurance along with decreased muscle damage [18]. The aims of this work were (1) to test whether ApN affords similar anti-inflammatory properties in human dystrophic myotubes, (2) to identify novel myokines targeted by ApN in these DMD myotubes, and (3) to unravel the more proximal events underlying the anti-inflammatory effects of ApN.

## Methods

### Primary culture of human myotubes

Primary human myoblasts from DMD patients (n = 4; age range: 11–31 year; biopsies taken from dorsal, paravertebral, *rectus femoris*, or *tensor fasciae latae* muscles) and Control (C) subjects (n = 3; 2 men and one woman; age range: 12–20 year; biopsies from dorsal/paravertebral, *tensor fasciae latae*, or quadriceps muscles) were provided by the French Telethon Myobank-AFM (Association Française contre les Myopathies).

To enhance myoblast enrichment, the cell suspension was first pre-plated on an uncoated Petri dish for 20 min to minimize contamination by fibroblasts, which preferentially adhere to the surface of the culture vessel [19]. Myoblasts were then recovered from the supernatant and seeded on a coated 75 cm^2^ cell culture flask at 37 °C in the presence of 5% CO_2_ in F-12 (Ham) supplemented with 20% fetal bovin serum (FBS), 1% L-glutamine (200 mM), and 100 µg/ml Primocin™ (Invivogen, Toulouse, France) (all other products from Life Technologies, Thermo Fisher Scientific, Erembodegen, Belgium) and grown until 70% confluence before trypsinization. Next, cells were seeded on 6-well plates at 5×10^4^ cells/well and allowed to proliferate for 5–6 days.

After the proliferation phase, the growth medium was replaced by the fusion medium, which consists of 1 part DMEM, 1 part F-12 (Ham), 2% horse serum (HS), 1% L-glutamine, and 1% Primocin™. Differentiation was allowed to continue for 11 days (time required to obtain mature myotubes) before the experimentation period. Cells were always used at passages between 4 and 10. We have usually generated at least two independent cultures (i.e., run at different times and for each time, from a new vial of cryopreserved myoblasts) from a given biological donor. The donors were always chosen at random to avoid any bias of selection.

### Inflammatory challenge and ApN treatment

Myotubes were challenged by human recombinant TNFα (Tumor necrosis factor alpha) (10 ng/ml) + IFNγ (Interferon gamma) (10 ng/ml) and/or ApN (5 µg/ml), for 24 h (TNFα from Tebu-Bio, Boechout, Belgium; IFNγ from R&D systems, Abington, UK; and ApN from Biovendor, Brno, Czech republic), as already described [18].

In some experiments, cells were first transfected before inflammatory challenge and/or ApN treatment [18]. Briefly, 5.10^4^ cells/well were transfected with either the On-Targetplus Non-targeting pool siRNAs (negative control, NT siRNAs), or a specific On-Targetplus siRNA SMARTpool against human AdipoR1 (50 nM) or human SIRT1 (50 nM) or human PGC-1α (70 nM) (all from Dharmacon, Thermo Fisher Scientific) using 7 µl Lipofectamine RNAiMAX reagent (Life Technologies) for 24 h. Next, the medium was renewed and cells were treated with TNFα + IFNγ with or without ApN for an additional 24 h.

At the end of the experiments, cells were rinsed twice in PBS before RNA extraction. When cytokine secretion had to be measured, culture media were also collected and stored at −20 °C for cytokine array/quantification. For a given culture, experiments were always performed in duplicate and the data obtained were then averaged.

### Cytokine arrays and ELISAs on myotubes-conditioned media

Screening for cytokines secreted by myotubes was performed by hybridizing medium with antibody-coated membranes according to the protocol supplied by the manufacturer [RayBio Human Cytokine Antibody Array C1000, a kit combining membranes of Arrays C6 and C7 and allowing the simultaneous detection of 120 cytokines (cat. no. AAH-CYT-1000, for details see http://www.raybiotech.com/files/manual/Antibody-Array/AAH-CYT-1000.pdf; RayBiotech, Tebu-bio)]. Briefly, 1 ml of medium was incubated with arrayed antibody supports for 5 h at room temperature; membranes were then washed and incubated with the mix of biotin-conjugated antibodies at 4 °C, overnight. After washing, horseradish peroxidase-conjugated streptavidin was added to the membranes for 2 h at room temperature. Spot intensities on membranes were quantified by scanning densitometry (Gel-Doc2000; Bio-Rad Laboratories, Mitry-mory, France) and analyzed with Image J program (National Institutes of Health, Maryland, USA). Background linked to non-conditioned medium (i.e., fusion medium with 2% HS incubated for 24 h) was subtracted from each sample. Signals were normalized to internal positive controls present on each membrane (see Fig. 4a) and then expressed as pixel density units.

The cytokines identified by arrays as differently (*P* < 0.1) secreted in the presence of ApN were further quantified by specific ELISAs: IL-11, interleukin-11; IL-17A, interleukin-7A; IL-6, interleukin-6; sgp130, soluble gp130 transducer chain; sTNFRII, soluble tumor necrosis factor receptor 2; IL-2Rα, interleukin-2 receptor alpha; GDNF, glial cell-derived neurotrophic factor; TPO, thrombopoietin; CCL28, Chemokine (C-C Motif) ligand 28, and MCP-1, monocyte chemiotactic protein-1, according to the protocol supplied by the manufacturer (all from RayBiotech). Moreover, we also measured TNFα (RayBiotech) by ELISA, which actually escaped detection by arrays, because of the known relationships between TNFα and ApN [20] and our past experience [the background generated by non-conditioned medium may mask the low amounts of some cytokines secreted by cultured cells (such as TNFα)] [21, 22].

ApN secreted into the medium, in basal conditions, was also measured by ELISA (Abcam, Cambridge, UK).

### Western blotting

Protein extracts were prepared using a lysis buffer (Cell Signaling Technology, BIOKE, Leiden, The Netherlands) supplemented with 1% protease inhibitor cocktail (Active Motif, Rixensart, Belgium). Forty micrograms of protein were dissolved in Laemmli buffer, subjected to SDS–PAGE under reducing and heat-denaturating conditions, and then transferred to PVDF membrane. Immunoblotting was performed using Utrophin (8A4) (1/200) (Santa Cruz, Heidelberg, Germany), according to the manufacturer’s instructions. Signals were revealed by enhanced chemiluminescence. Band intensities were quantified by scanning densitometry and analyzed with Image J program, as described earlier. Signals were then normalized to Ponceau band intensity.

### RNA extraction and real-time quantitative PCR (RT-qPCR)

RNA was isolated from cultured cells with TriPure reagent (Roche Diagnostics, Vilvoorde, Belgium). One microgram of total RNA was reverse transcripted using RevertAid H Minus First-Strand cDNA Synthesis Kit (Thermo Scientific). RT-qPCR primers for human Tata box-binding protein (TBP), ApN, TNFα, IL-6, and Utrophin A (UTRN A) were similar to those previously reported [18, 23]. The other primer sequences were IL-17A (sense, 5′-AACGCTGATGGGAACGTGGA-3′; antisense, 5′-GCAGCCCACGGACACCAGTA-3′), CCL28 (sense, 5′-GGATTGTGACTTGGCTGCTGTC-3′; antisense, 5′-CCATGGTGTTTCTTCCTGTGGC-3′) MyoD (sense, 5′-CTGCTCCTTTGCCACAACG-3′; antisense, 5′-GAGTCGAAACACGGGTCGTC-3′), Myogenin (sense, 5′-ACTTCTACCAGGAACCCCGCT-3′; antisense, 5′-GGACAGGCAGGTAGTTTTCCC-3′), Mrf4 (sense, 5′-TAACGGCTAAGGAAGGAGGAGC-3′; antisense, 5′-CAAGCGCAGGCTCAGTTACTTC-3′), AdipoR1 (sense, 5′-ACTCCTAAGCACCGGCAGAC-3′; antisense, 5′-CAAGCCAAGTCCCAGGAACA-3′), SIRT1 (sense, 5′-AACAGGTTGCGGGAATCCAAAG-3′; antisense, 5′-GGCACCTAGGACATCGAGGAAC-3′) and PGC-1α (sense, 5′-TTGACTGGCGTCATTCAGGAGC-3′; antisense, 5′-AGGAAGATCTGGGCAAGAGGC-3′). 40–80 ng total RNA equivalents were amplified using an iCycler iQ real-time PCR detection system (Bio-Rad Laboratories) [23]. The threshold cycles (Ct) were measured in separate tubes and in duplicate. The analysis of the melting curve was carried out at the end of the amplification. To ensure the quality of the measurements, each plate included a negative control for each set of primers. ΔCt values were calculated in every sample for each gene of interest as follows: Ct _gene of interest_−Ct _reporter gene_, with TBP as the reporter gene. Relative changes in the expression level of one specific gene (ΔΔCt) were calculated as ΔCt of the test group minus ΔCt of the reference group and then presented as 2^−ΔΔCt^.

### Result presentation and statistical analysis

Results are means ± SEM for the indicated number of independent cultures. Comparisons between the two groups of subjects (C versus DMD) were carried out using two-tailed unpaired Student’s *t* test. Comparisons between different conditions within a given experiment were made using two-tailed paired Student’s *t* test (two conditions) or repeated analysis of variance (several conditions) with Bonferroni post-test correction (Prism 5; GraphPad Software, California, USA). Differences were considered statistically significant at *P* < 0.05.

For the screening by cytokine antibody arrays, the differences that were statistically significant (*P* < 0.05) and those that were “borderline” (*P* < 0.1) were taken into account for subsequent ELISA analysis to avoid false negatives due to the low number of independent cultures used in this screening.

## Results

### Characterization of human control and DMD myotubes

**Fig. 1:**
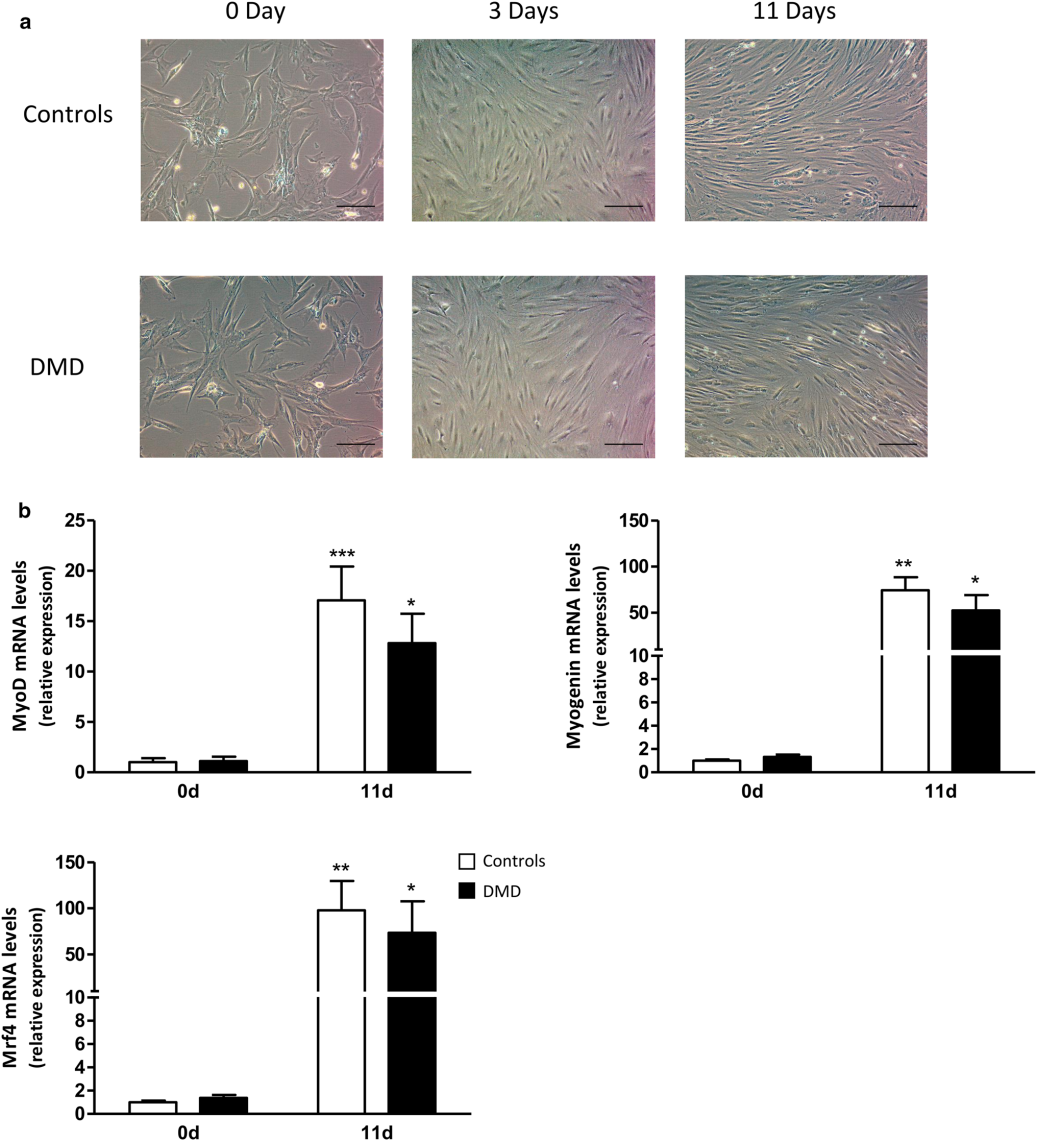
Characterization of human control and DMD myotubes. **a** Representative phase-contrast microscopy images of human control and DMD cells cultured in the differentiation medium for 0, 3, or 11 days. *Scale bar* 100 µm. **b** RNA levels of myogenic markers before (day 0) and after 11 days of differentiation. mRNA levels were normalized to TBP. The subsequent ratios are presented as relative expression compared to C myotubes at 0 day. Data are means ± SEM; *n =* 5–7 independent cultures from 3 to 4 different patients per group. *p < 0.05, **p < 0.01, ***p < 0.001 for day 11 versus day 0

DMD myoblasts differentiated at the same extent as control ones, in agreement with a previous report [24]. We confirmed these data by phase-contrast microscopical examination and expression of myogenic markers. Differentiation of myoblasts into myotubes did not differ between control (C) and DMD cells, as assessed microscopically at day 0, 3, and 11 after initiation of differentiation (i.e., before, at intermediate, and late stages of differentiation) (Fig. 1a). More specifically, at day 11, mature myotubes exhibited the same characteristic elongated morphology and a similar degree of fusion in both groups. Likewise, gene expression of myogenic markers increased similarly in C and DMD cells during the differentiation process (Fig. 1b). We studied 3 markers: MyoD, known to commit cells to the myogenic program, and Myogenin and Mrf4 involved later in the differentiation phase itself [25].

### Basal production of adiponectin by human control and DMD myotubes

**Fig. 2:**
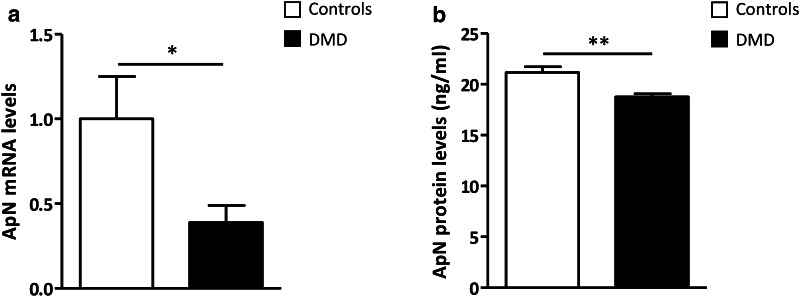
Adiponectin production by myotubes from control and DMD subjects. **a** mRNA levels, measured in myotubes, were normalized to TBP. The subsequent ratios are presented as relative expression compared to C myotubes. **b** ApN secreted in medium for 24 h was quantified by ELISA and expressed in ng/ml. Data are means ± SEM; n = 6 independent cultures from 3 to 4 different patients per group. *p < 0.05, **p < 0.01 for DMD versus C subjects

We examined the production of ApN in the basal state. Compared to controls, ApN mRNA and secretion from dystrophic myotubes were decreased by ~60% and ~15%, respectively (Fig. 2a, b). This suggests a potential deficiency of ApN in DMD myotubes and supports the rationale behind recommending ApN supplementation in muscle dystrophy.

### Anti-inflammatory properties of adiponectin in human control and DMD myotubes

We then tested whether ApN treatment retained its anti-inflammatory properties in dystrophic muscle. To this end, we investigated if this hormone could reverse the pro-inflammatory pattern of cytokine expression in myotubes from DMD patients.

**Fig. 3:**
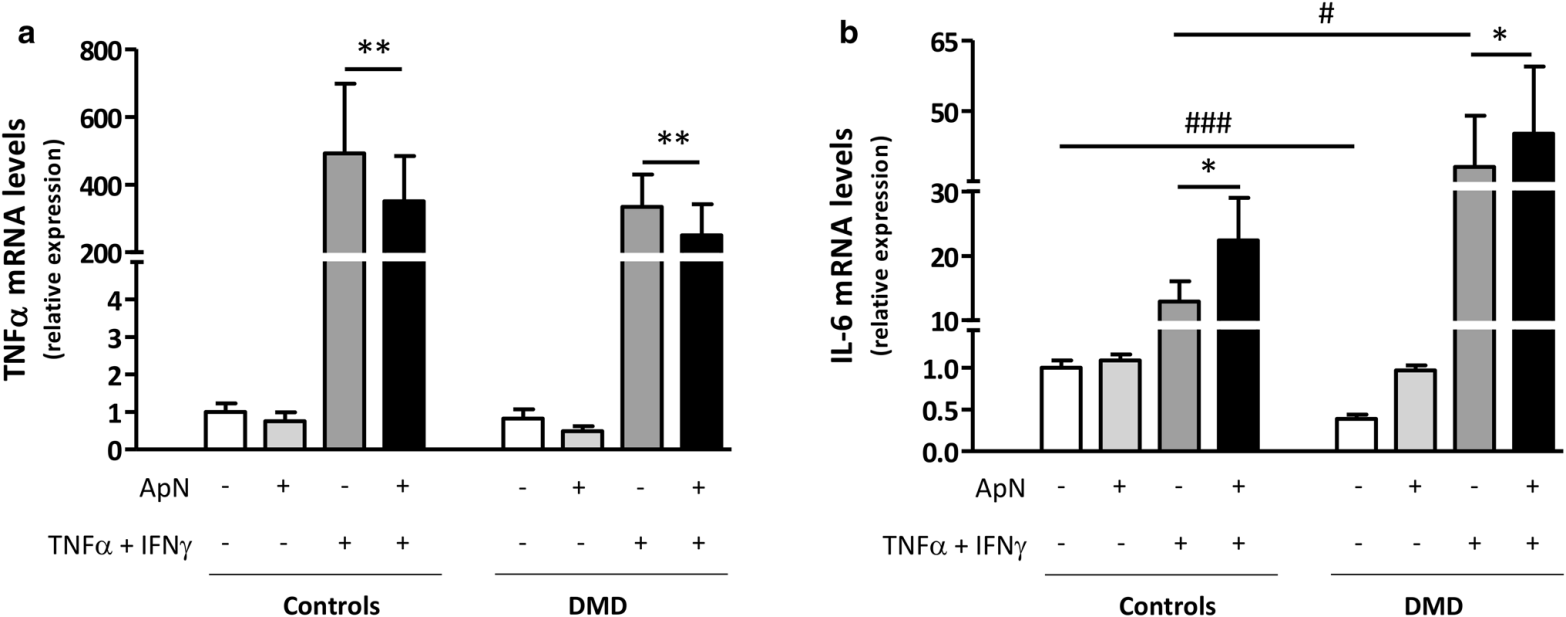
Effects of adiponectin on inflammatory markers in myotubes from control and DMD subjects. TNFα (**a**) and IL-6 (**b**) mRNA levels in human C and DMD myotubes, which were challenged by an inflammatory stimulus (a combination of TNFα and IFNγ), while being or not treated with ApN. mRNA levels were normalized to TBP. The subsequent ratios are presented as relative expression compared to basal conditions (no TNFα/IFNγ and no ApN) in C subjects. Data are means ± SEM; n = 8–10 independent cultures (i.e., run at different times and for each time, from a new vial of cryopreserved myoblasts) from 3 to 4 different subjects in each C and DMD group. The subjects were always chosen at random. Comparisons between different conditions were made using repeated analysis of variance with Bonferroni post-test correction. *p < 0.05, **p < 0.01 for the respective conditions with versus without ApN. ^#^ *p* < 0.05, ^###^ *p* < 0.01 for the respective conditions in DMD versus C

**Fig. 4:**
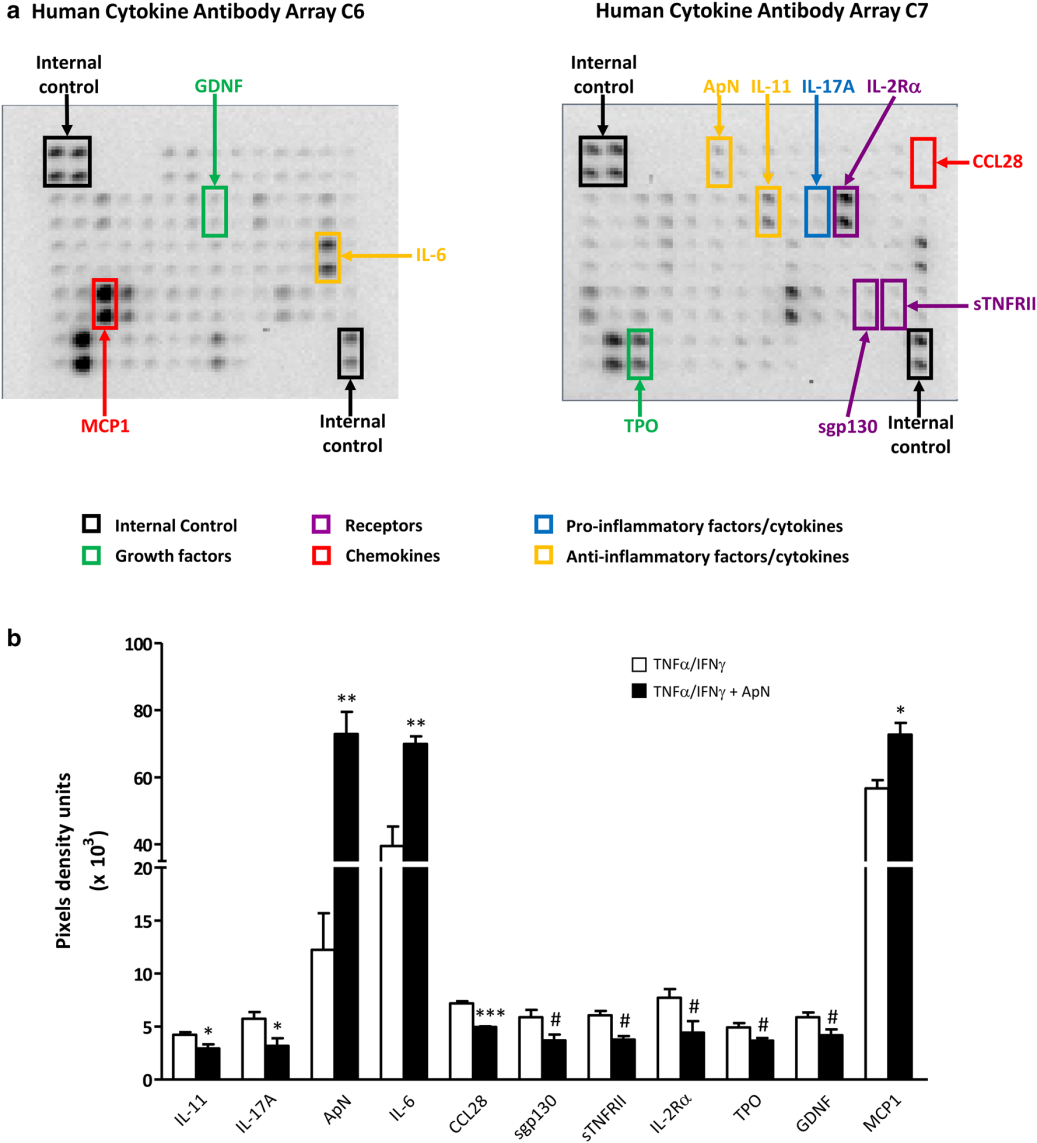
Screening secretome of myotubes from DMD patients. DMD myotubes were challenged by an inflammatory stimulus (a combination of TNFα and IFNγ), while being or not treated by ApN. A set of 2 Raybiotech membranes, which tests together 120 cytokines, was probed with conditioned-media to detect myokine secretion. **a** This figure illustrates a representative array of conditioned-media, after the inflammatory challenge but without ApN treatment. Highlighted myokines represent the factors, which turned out to be modified by ApN treatment. Each color corresponds to a specific factor family. **b** Cytokine levels were measured by chemiluminescence, normalized to internal positive controls, and expressed as pixel density units. Only the myokines, which were (or tended to be) modified by ApN, are shown in the histograms. Three myokines were more abundantly secreted, while 8 others were or tended to be less secreted after ApN treatment. Values are means ± SEM; n = 3 independent cultures, each from one different DMD patient. IL-11, interleukin-11; IL-17A, interleukin-17A; IL-6, interleukin-6; ApN, adiponectin; sgp130, soluble gp130 transducer chain; sTNFRII, soluble tumor necrosis factor receptor 2; IL-2Rα, interleukin-2 receptor alpha; GDNF, glial cell-derived neurotrophic factor; TPO, thrombopoietin; CCL28, chemokine (C-C Motif) ligand 28; and MCP-1, monocyte chemiotactic protein-1. *p < 0.05, **p < 0.01, ***p < 0.001 for the effects of ApN. Differences, which tended to be but were not statistically significant, are also indicated in this figure (^#^ *p* < 0.10) and were considered for subsequent ELISA analysis

To mimic the inflammatory microenvironment, which prevails in DMD, we challenged the myotubes by an inflammatory stimulus (TNFα/IFNγ), TNFα playing a key pathogenic role in worsening the disease [18]. In C myotubes, TNFα combined with INFγ induced its own gene expression and that of IL-6, the latter being viewed as an attempt to counteract the inflammatory state (Fig. 3a, b, 3rd histograms) [26]. Concomitant ApN treatment downregulated gene expression of TNFα (~−30%) and further upregulated that of IL-6 (~+75%) (Figs. 3a, b, 4th histograms). TNFα gene expression behaved similarly in C and DMD myotubes (Fig. 3a). Compared to controls, basal gene expression of IL-6 was blunted in DMD myotubes (~−60%), which could possibly result from low ApN, while its inflammatory response was enhanced (+300%) (Fig. 3b; compare the two white columns and the two dark gray ones). Yet, the anti-inflammatory effects of ApN were preserved in DMD myotubes: the hormone actually downregulated TNFα mRNAs (~−25%) while further upregulating IL-6 mRNAs (~+20%) (Fig. 3a, b; compare the last two columns of each panel). These data suggest that ApN may retain its anti-inflammatory properties in dystrophic muscle.

### Myokine secretion profiling and identification of novel adiponectin targets in DMD myotubes

Media from DMD myotubes, which have been challenged by the inflammatory stimulus in the presence or in the absence of ApN treatment, were next screened by cytokine antibody arrays (Fig. 4a). Among the 120 cytokines tested, 64 were secreted by myotubes. Ten were or tended to be differently secreted in the presence of ApN (*P* < 0.1 or less; Fig. 4a): eight cytokines were hypo-secreted, while two others were over-secreted (ApN, which was artificially increased by the treatment was excluded *a posteriori* from subsequent analysis) (Fig. 4a, b). These myokines belong to five families: pro- and anti-inflammatory factors/cytokines, growth factors, soluble receptors, and chemokines.

**Fig. 5:**
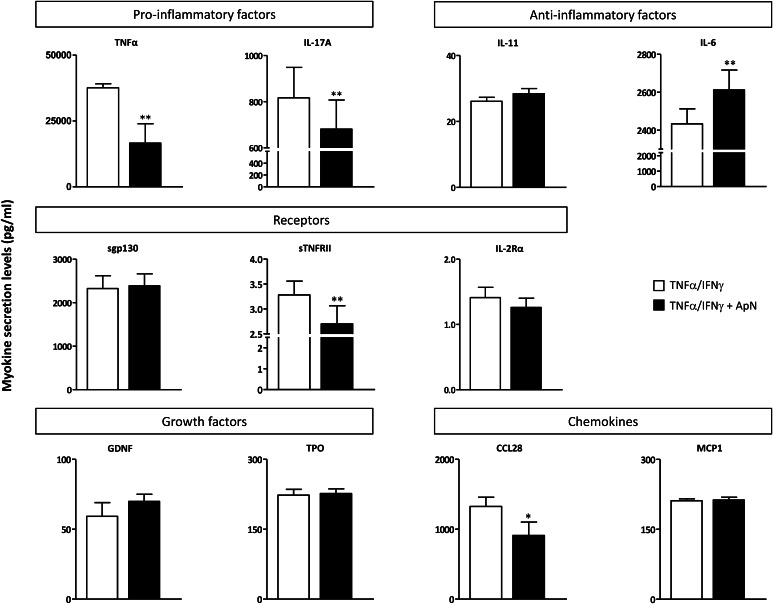
Effects of adiponectin on myokine secretion by human DMD myotubes. Quantification of myokine secretion by DMD myotubes challenged by an inflammatory stimulus (a combination of TNFα and IFNγ), while being or not treated with ApN. Myokines, which were (or tended to be) modified in the presence of ApN, were first identified by cytokine antibody arrays (see Fig. 4a, b). Afterward, myokine concentrations in culture medium were quantified by specific ELISAs and expressed in pg/ml. Data are means ± SEM; n = 5–7 independent cultures from 4 different patients. *p < 0.05, **p < 0.01 for the effect of ApN

These myokines were next quantified by specific ELISAs (Fig. 5). TNFα was also measured by this technique (see Methods). The secretion of two pro-inflammatory factors (TNFα and IL-17A), one soluble receptor (sTNFRII), and one chemokine (CCL28) was downregulated by ApN treatment (Fig. 5). Conversely, only one myokine was upregulated by ApN: IL-6, which may exhibit anti-inflammatory properties (Fig. 5).

### Gene expression of myokines in control and DMD myotubes

**Fig. 6:**
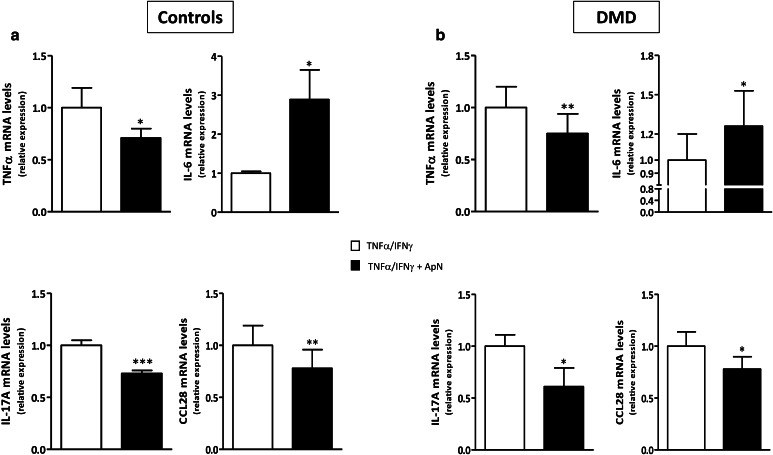
Effects of adiponectin on myokine gene expression in human control (**a**) and DMD (**b**) myotubes. mRNA levels of the myokines, which were found to be modified by ELISA in Fig. 5, are shown here. As there is no specific transcript for sTNFRII, gene expression of this soluble receptor could not be measured. For the other myokines, mRNAs were quantified by RT-qPCR in C **(a)** and DMD **(b)** myotubes challenged by an inflammatory stimulus, while being or not treated with ApN. mRNA levels were normalized to TBP and presented as relative expression compared to the respective control conditions (i.e., with TNFα/IFNγ and no ApN). Results are means ± SEM; n = 6–8 independent cultures from 3 to 4 different patients in each C and DMD group. *p < 0.05, **p < 0.01, ***p < 0.001 for the effect of ApN

To investigate whether these changes in myokine secretion were in part mediated by pretranslational mechanisms, we quantified gene expression in DMD myotubes. C myotubes were used for comparison. sTNFRII could not be tested, as there are no specific transcripts to discriminate between the soluble and non-soluble forms of this receptor. mRNA levels of TNFα, IL-17A, and CCL28 were decreased, whereas those of IL-6 were increased in both C and DMD myotubes (Fig. 6). Hence, the four myokines exhibited a pattern of secretion roughly similar to that of mRNA abundance (Compare Figs. 5, 6), thereby indicating a pretranslational regulation for these changes.

### Mechanisms underlying the beneficial effects of adiponectin in control and DMD myotubes

We next investigated whether the signaling pathway linking AdipoR1-SIRT1-PGC-1α was involved in the anti-inflammatory action of ApN in DMD myotubes, as previously shown in healthy myotubes [18]. To this end, we knocked down each component of this pathway using specific siRNAs.

**Fig. 7:**
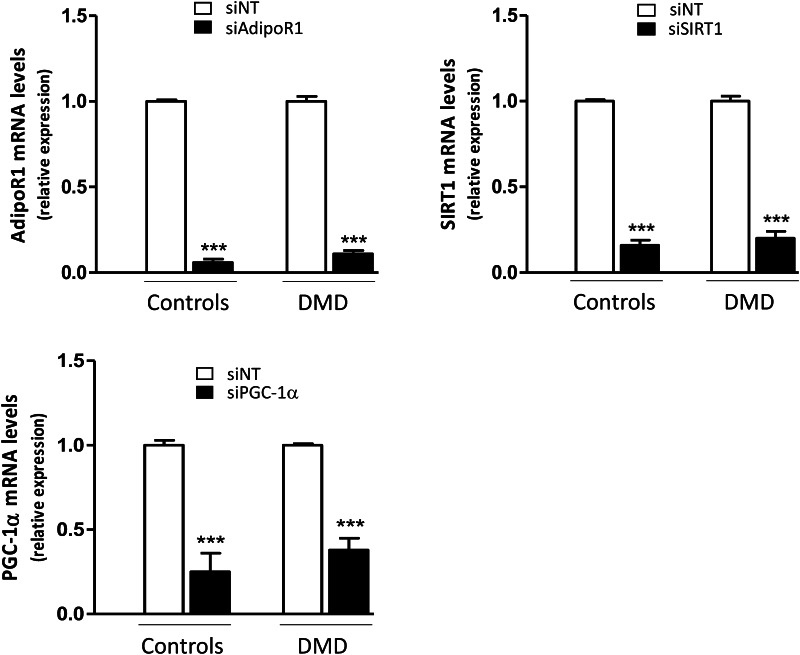
Efficiency of gene silencing by siRNA in human control and DMD myotubes. Control and DMD myotubes were transfected with siRNAs against human AdipoR1, SIRT1, or PGC-1α or a negative (non-targeting, siNT) control, for 24 h. After transfection, cells were challenged with TNFα/IFNγ for 24 h. mRNA levels were normalized to TBP and presented as relative expression compared to control conditions (i.e., siNT). The results presented herein are means ± SEM; n = 6 independent cultures from 3 to 4 different patients for each group. AdipoR1, adiponectin receptor type 1; SIRT1, sirtuin1; PGC-1α, peroxisome proliferator-activated receptor-coactivator-1 alpha. ***p < 0.001 for the effects of siRNA

**Fig. 8:**
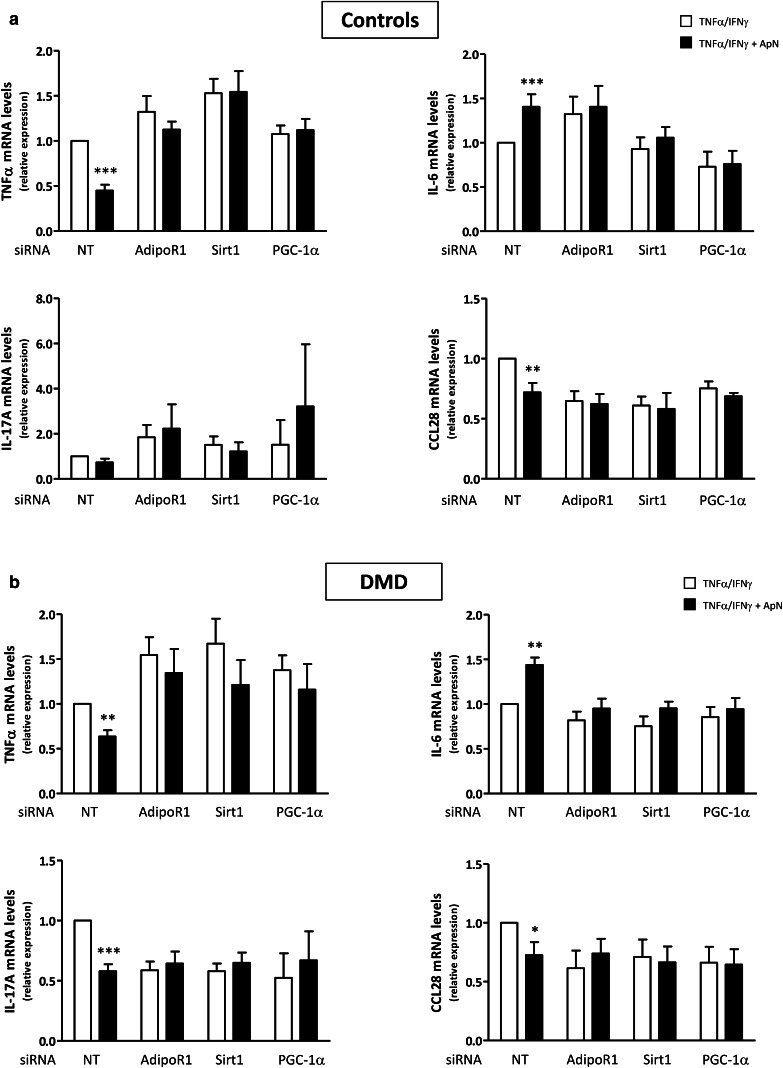
Implication of the AdipoR1/SIRT1/PGC-1α pathway in the anti-inflammatory effects of adiponectin in control (**a**) and DMD (**b**) myotubes. Myotubes were transfected with siRNAs against AdipoR1, SIRT1, PGC-1α, or a negative (non-targeting, NT) control. After transfection, cells were challenged with TNFα/IFNγ, while being or not treated with ApN, as described above. mRNA levels of TNFα, IL-17A, IL-6, and CCL28 were normalized to TBP and presented as relative expression compared to control conditions (i.e., NT siRNA without ApN). The results presented herein are means ± SEM; *n* = 7–10 independent cultures from 3 to 4 different patients for each group. AdipoR1, adiponectin receptor type 1; SIRT1, sirtuin1; PGC-1α, peroxisome proliferator-activated receptor- coactivator-1 alpha. *p < 0.05, **p < 0.01, ***p < 0.001 for the effects of ApN

We first confirmed that siRNA silencing was efficient as shown by marked decreases of target gene expression (−65 to −95%) (Fig. 7). Next, we demonstrated that in C myotubes, ApN exerted its anti-inflammatory action in basal conditions (i.e., in the presence of non-targeting (NT) siRNAs): it reduced TNFα (-55%) and CCL28 (–28%) mRNAs, while increasing IL-6 mRNAs (+40%) [Compare the first two histograms (black versus white column) of each panel; Fig. 8a]. In this experiment, ApN effect on IL-17A was not evidenced for unclear reasons. The anti-inflammatory effects of ApN on all the other myokines were abolished by siRNA silencing of genes encoding for either AdipoR1, SIRT1, or PGC-1α (Fig. 8a).

In DMD myotubes, ApN similarly decreased mRNA levels of TNFα (−37%) and CCL28 (−28%), and also of IL-17A (−42%), while upregulating IL-6 mRNA levels (+44%) in basal conditions [when the cells were transfected with NT siRNAs: compare the first two histograms (black versus white column) of each panel from Fig. 8b]. These changes were again abrogated when the myotubes were transfected with siRNAs against AdipoR1, SIRT1, or PGC-1α.

Thus, each component of the AdipoR1-SIRT1-PGC-1α signaling pathway appears to be necessary for the anti-inflammatory action of ApN in both C and dystrophic muscle.

**Fig. 9:**
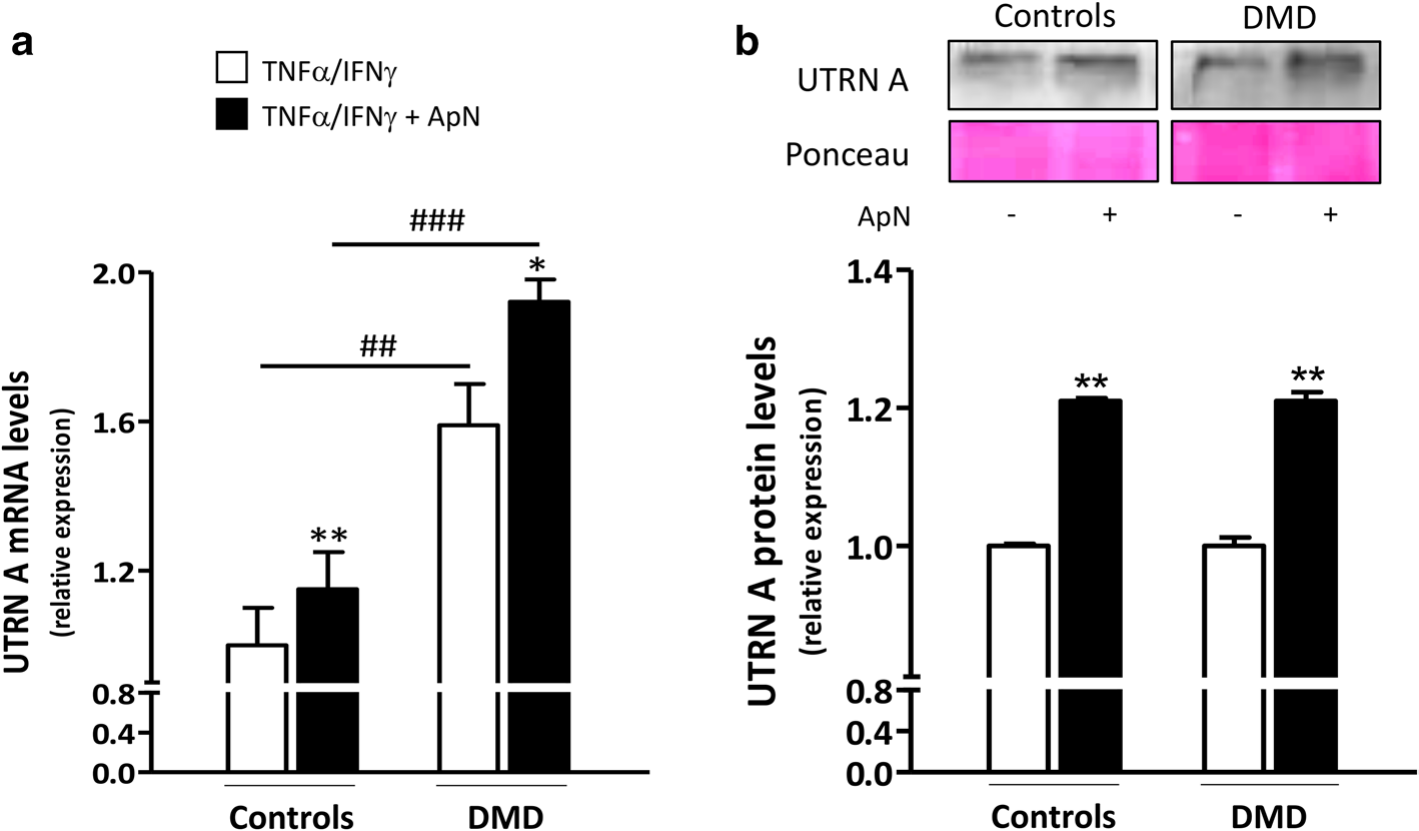
Effects of adiponectin on utrophin A mRNA and protein levels in human control and DMD myotubes. Utrophin A (UTRN A) mRNA and protein levels were measured in C and DMD myotubes challenged by an inflammatory stimulus (with a combination of TNFα and IFNγ) and treated or not with ApN. **a** mRNA levels were normalized to TBP and presented as relative expression compared to control conditions (i.e., TNFα/IFNγ without ApN in C). **b** Protein levels on a representative Western Blot and Ponceau stain. Data from Western blots were quantified and normalized to Ponceau. The subsequent ratios are presented as relative expression compared to control conditions. Results are means ± SEM; n = 5–6 (**a**) and 3–6 (**b**) independent cultures from 3 to 4 different patients for each group. *p < 0.05, **p < 0.01 for the effect of ApN; ^##^ *p* < 0.01, ^###^ *p* < 0.001 for DMD versus C subjects

Because PGC-1α is a transcriptional coactivator, it has the potential to alter the expression of numerous genes. More specifically, it can induce utrophin A, an autosomal analog of dystrophin [27]. In agreement with a previous report [28], utrophin A gene expression was more abundantly expressed in DMD than in C myotubes (+60%), likely as an attempt to compensate the lack of dystrophin (Fig. 9a). Yet, we were not able to show an increase in protein levels in our acutely inflamed cells (Fig. 9b). Consistent with our working hypothesis on ApN action, utrophin A mRNAs were upregulated by ApN in both C (+15%) and DMD myotubes (+21%) and the protein levels showed a similar upregulation (+21% in both groups) (Fig. 9a, b).

## Discussion

Muscle inflammation plays a crucial role in DMD pathogenesis [2]. We first found a decrease in ApN production by human dystrophic myotubes. We further showed that ApN supplementation retains its anti-inflammatory properties in these DMD myotubes. We eventually deciphered the underlying mechanisms by investigating alterations in the myokine secretion profile as well as in more proximal events of the signaling cascade.

First, basal ApN expression and secretion of ApN were decreased in dystrophic myotubes compared to controls. We have previously shown that in an environment of acute or chronic inflammation, ApN was overproduced in vivo by the “normal” mouse skeletal muscle to locally counteract excessive and deleterious inflammatory reactions [29, 30]. By contrast, the skeletal muscle of mdx mice was unable to overproduce local ApN {[18], data not shown}. Likewise, this ability seems to be lost in human dystrophic myotubes, where ApN production was even decreased when compared to that of controls. This suggests a potential deficiency of ApN in DMD myotubes and supports the rationale behind recommending ApN supplementation in this pathology.

The effects of ApN on the myokine secretory profile are still largely undescribed. Moreover, the secretome profile has been studied in mouse dystrophic (mdx) myotubes only [3], but not yet in human dystrophic ones. We thus analyzed the secretome profile of DMD myotubes before or after ApN treatment. Using cytokine antibody array and ELISA approaches, we identified several myokines as newly secreted by human dystrophic myotubes and regulated by ApN. These myokines belong to four families: pro- and anti-inflammatory factors/cytokines, soluble receptors, and chemokines, thereby indicating that the muscle fibers themselves contribute to modulate and possibly to actively perpetuate the inflammatory process [2]. ApN downregulated several molecules promoting inflammation (TNFα, IL-17A, and CCL28), while upregulating IL6, which exert a dual role in immune responses. To our knowledge [11, 31], two of these molecules (sTNFRII, CCL28) are novel secretory products of human myotubes.

In our study, ApN downregulated TNFα expression and secretion in DMD myotubes like in controls. Circulating TNFα is markedly increased in DMD subjects [32] and its expression is upregulated in dystrophic muscle [2]. Anti-TNFα therapy administered to very young mdx mice for 3 weeks prevented muscle damage [33]. sTNFRII, a soluble form of TNFα receptor, acting as a cytokine inhibitor [34] was also reduced under ApN treatment. This reduction is likely to result from the concomitant decrease of TNFα. It is of note that a reverse situation was observed after the administration of TNFα to humans: higher systemic levels of sTNFRII were produced [35]. Besides TNFα, ApN also downregulated two other pro-inflammatory myokines: one interleukin, IL-17A, and one chemokine, CCL28. IL-17A is a potent amplifier of ongoing inflammation, which plays an important role in the progression of chronic inflammation and autoimmunity [2]. Recently, ApN has been found to suppress IL-17A production from T cells, thereby attenuating psoriasiform skin inflammation [36]. This suppressive effect is of importance in our own study since muscle IL-17A mRNA levels have been reported to be higher in DMD than that in non-DMD subjects and to be associated with the clinical outcome of the patients [37]. ApN also downregulated the pro-inflammatory chemokine CCL28, which belongs to the CC chemokine family. CCL28 is highly expressed by epithelial cells that line the mucosa and directs the migration of plasma cells to these sites [38]. As yet, the presence and function of CCL28 have not been documented in skeletal muscle. In contrast to the other myokines, which were downregulated by ApN, the production of IL-6 was upregulated in both control and DMD myotubes. IL-6 is not only involved in inflammation and infection responses, but also in the regulation of regenerative and anti-inflammatory processes, by acting through two signaling modes [39]. IL-6 is expressed at high levels in the serum of DMD boys as well as in inflammatory cells infiltrating their tissues [40]. On one hand, IL-6 blockade with an antibody neutralizing the IL-6 receptor attenuated the dystrophic phenotype of mdx mice [40]. On the other hand, in another report, IL-6 blockade increased muscle inflammation without improving muscle function, thereby suggesting a potential anti-inflammatory role for IL-6 in mdx mice [26]. The two studies differed from dosing regimens [41], age of mice at initiation of injections, and treatment duration (2 weeks for the beneficial versus 5 weeks for the deleterious effects). In line with the anti-inflammatory properties of IL-6, which were also evidenced after muscle exercise [5], this myokine has further been reported to play a beneficial role in muscle regeneration by promoting myoblast differentiation [42]. Because of the potent anti-inflammatory and regenerative properties of ApN in mdx mice, we hypothesize that IL-6 could be one mediator of these effects. Taken together, our data suggest that ApN regulates the secretion of downstream myokines, thereby inducing a shift in the immune balance of DMD myotubes toward a less inflammatory phenotype.

After identifying these myokines, we examined whether their regulation occurred, at least in part, at the pretranslational level. We thus quantified gene expression. Most myokines exhibited a similar pattern between mRNA abundance and secretion levels, indicating a pretranslational effect of ApN. Besides, these effects were roughly qualitatively similar in control and DMD subjects.

To unravel, in more details, the mechanisms underlying the anti-inflammatory effects of ApN on dystrophic muscle, we explored earlier events of the ApN signaling cascade. We have previously shown that ApN mediates its protection on control myotubes via AdipoR1 and the AMPK-SIRT1-PGC-1α pathway, thereby leading to Nuclear factor-kappa B (NF-κB) repression [18]. Here, silencing of selected genes (AdipoR1, SIRT1, or PGC-1α) further abrogated ApN action on downstream myokines and this inhibition occurred in both control and DMD myotubes. Hence, each of these silenced components appears, therefore, to be necessary for ApN action on the myokine profile. Because the same signaling pathway is involved in C and DMD myotubes, it is also likely that the repression of NF-κB contributes to the anti-inflammatory effects of ApN in dystrophic muscle.

Because PGC-1α is a transcriptional coactivator [43], it has the potential to alter the expression of numerous genes including utrophin A, an autosomal homolog of dystrophin [43]. Herein, in basal conditions, utrophin A was more expressed in DMD than in control myotubes, likely to compensate the lack of dystrophin [44]. Several studies have indeed reported that utrophin A can restore sarcolemma integrity and serve as a surrogate to dystrophin in dystrophic muscle [27]. As already shown in controls [18], ApN upregulated utrophin A in DMD myotubes, which may also contribute to rescue the dystrophic phenotype.

The only medications so far shown to be effective in delaying the progression of DMD are glucocorticoids [45]. Their beneficial effects are mostly ascribed to reduced inflammation [46]. However, adverse effects must be considered: weight gain, growth retardation, and cushingoid appearance are described as well as fractures, hypertension, and glucose intolerance [47, 48]. ApN could well be a strong alternate candidate for DMD therapy since besides its potent anti-inflammatory properties, it also protects against most common side effects of glucocorticoids such as obesity, hypertension, and glucose intolerance [13], while further improving the myogenic program [18, 49, 50] and upregulating utrophin A. Some novel myokines identified as regulated by ApN in DMD myotubes, like IL-17A or CCL28 may also be additional new targets for the management of this disease.

In conclusion, ApN retains its beneficial properties in dystrophic muscles by activating AdipoR1 and the AMPK-SIRT1-PGC-1α pathway, thereby inducing a shift in the secretion of downstream myokines toward a less inflammatory profile while upregulating utrophin A. ApN as well as its downstream myokines may be therapeutic targets for the management of DMD.

## Abbreviations

DMD: Duchenne Muscular Dystrophy
ApN: Adiponectin
AdipoR1: Adiponectin receptor 1
AdipoR2: Adiponectin receptor 2
AMPK: AMP-activated protein kinase
PPARα: Peroxisome proliferator-activated receptor alpha
SIRT1: Sirtuin1
PGC-1α: Peroxisome proliferator-activated receptor-γ coactivator-1α
TNFα: Tumor necrosis factor alpha
IFNγ: Interferon gamma
IL-6: Interleukin-6
IL-17A: Interleukin-17A
sTNFRII: Soluble tumor necrosis factor receptor 2
CCL28: Chemokine (C-C Motif) ligand 28
C: Control
NT: Non-targeting
NFκB: Nuclear factor-kappa B
AFM: Association Française contre les Myopathies
FBS: Fetal bovin serum
HS: Horse serum
IL-11: Interleukin-11
sgp130: Soluble gp130 transducer chain
IL-2Rα: Interleukin-2 receptor alpha
GDNF: Glial cell-derived neurotrophic factor
TPO: Thrombopoietin
MCP-1: Monocyte chemiotactic protein-1
TBP: Tata box-binding protein
UTRN A: Utrophin A
MyoD: Myogenic differentiation 1
Mrf4: Myogenic regulatory factor 4
